# Influence of Different Spot Pattern Lasers on Cleaning Effect of TC4 Titanium Alloy

**DOI:** 10.3390/ma18010061

**Published:** 2024-12-27

**Authors:** Xinqiang Ma, Tengchao Liu, Yuan Ren, Yanlu Zhang, Zifa Xu, Wei Cheng, Zhenzhen Zhang, Yongmei Zhu, Qinhe Zhang

**Affiliations:** 1 Key Laboratory High Efficiency & Clean Mech Manufacture, School of Mechanical Engineering, Shandong University, Ministry of Education, 17923 Jingshi Rd., Jinan 250061, China; maxinqiang@sdlaser.cn; 2 Laser Institute, Qilu University of Technology (Shandong Academy of Sciences), Jinan 250104, China; 3 Shandong Qiangyuan Laser of SDIIT Ltd., Liaocheng 252000, China; 4 School of Instrumentation and Optoeletronic Engineering, Beihang University, Beijing 100191, China

**Keywords:** Gaussian spot, Flat-top spot, numerical simulation, micro-morphology, element content

## Abstract

This study employed different spot pattern lasers to clean the oxide film on the surface of a TC4 titanium alloy. The variation in temperature field and ablation depth during the laser cleaning process was simulated by establishing a finite element model. The effects of various laser processing parameters on the micromorphology, elemental composition, and surface roughness of the TC4 titanium alloy were analyzed. The results show that as the laser energy density increases, both the temperature field and ablation depth increase as well. Under optimal laser processing parameters, the laser energy density is 5.27 J/cm^2^, with a repetition frequency of 300 kHz and a scanning speed of 6000 mm/s. A comparison of the cleaning effects of Gaussian pulse lasers and Flat-top pulse lasers reveals that the Gaussian pulse laser causes less damage to the TC4 titanium alloy, resulting in lower oxygen content and roughness values after cleaning compared to Flat-top pulse laser cleaning.

## 1. Introduction

TC4 titanium alloys exhibit outstanding properties, such as corrosion resistance, high-temperature resistance, low density, and high strength, making them prevalent in aerospace, petrochemical, and defense industries [1,2,3]. Owing to their high oxygen affinity, dense oxide films quickly form on titanium alloys during prolonged storage and thermal forming processes [4]. The presence of this oxide film can significantly reduce the fatigue strength, load-bearing capacity, and service life of the substrate [5,6]. Therefore, effectively removing the oxide film is crucial for titanium alloys’ subsequent processing and utilization. Traditional industrial cleaning methods could be more efficient and lead to environmental pollution. Laser cleaning uses the high energy level of lasers to remove contaminants through principles such as photodegradation and photo disruption. It features green, environmentally friendly, and efficient attributes, becoming an essential green processing technology in industrial cleaning [7,8].

The distinction between different spot patterns lies in the energy distribution of the laser spot, which can be categorized into Gaussian pulse lasers and Flat-top pulse lasers. Gaussian pulse spots have a Gaussian distribution, with energy concentrated at the center and less energy at the edges, making them suitable for high-precision processing and microstructural treatments [9]. In contrast, Flat-top pulse lasers distribute pulse energy uniformly across the entire Flat-top area, resulting in a circular Flat-top shape. This uniform energy distribution is beneficial for applications where it reduces the unevenness of the heat-affected zone during processing [10].

Currently, scholars, both domestically and internationally, have conducted extensive research on the cleaning of titanium alloys. Li et al. studied the cleaning of an oxide film on a TA15 titanium alloy by varying the laser processing parameters and reported that the removal effect is optimal when the oxide film temperature is marginally above its boiling point, with the removal mechanism being laser ablation [11]. Kumar et al. used nanosecond pulse lasers to clean Ti-3Al-2.5V tubes, experimentally determined the cleaning and damage thresholds, and found no defects during the subsequent welding of the cleaned tubes [12]. Wang et al. investigated the cleaning of a TC2 titanium alloy at various scanning speeds and reported that an optimal speed of 2500 mm/s yielded the best oxide film removal. As the scanning speed decreased, the surface roughness improved, and hardness increased [13]. Li employed a liquid-assisted method for cleaning titanium alloys and identified removal mechanisms, including laser ablation, laser-induced waves, and phase explosion, providing an effective surface-cleaning approach [14]. Turner et al. conducted laser cleaning on titanium alloy parts in aerospace engines, followed by electron beam welding, and noted a significant improvement in weld quality after laser cleaning [15]. Ragusich et al. utilized femtosecond and excimer lasers to strip a TiAlN corrosion-resistant coating from Ti-6Al-4V alloy surfaces, achieving effective removal, but stated that the femtosecond laser’s efficiency was inferior to that of the excimer laser [16]. Liu et al. studied the effects of energy densities on the surface morphology of a TA15 titanium alloy and reported that laser cleaning transformed the natural morphology into uniform pits, with grain refinement in the remelted layer enhancing the wear resistance [17].

In response to the application needs for the laser cleaning of TC4 titanium alloy, this study employs Gaussian pulse lasers and Flat-top pulse lasers to clean the oxide film on the surface of the TC4 titanium alloy. The removal effectiveness of the oxide film is analyzed through factors such as micromorphology, elemental composition, and surface roughness. A cleaning model is also established via COMSOL Multiphysics 6.2 to investigate the effects of different spot pattern laser processing parameters on the temperature field and ablation depth. By combining theoretical modeling with experimental methods, this research provides a basis for practical applications in subsequent endeavors.

## 2. Materials and Methods

### 2.1. Experimental Materials

**Figure 1:**
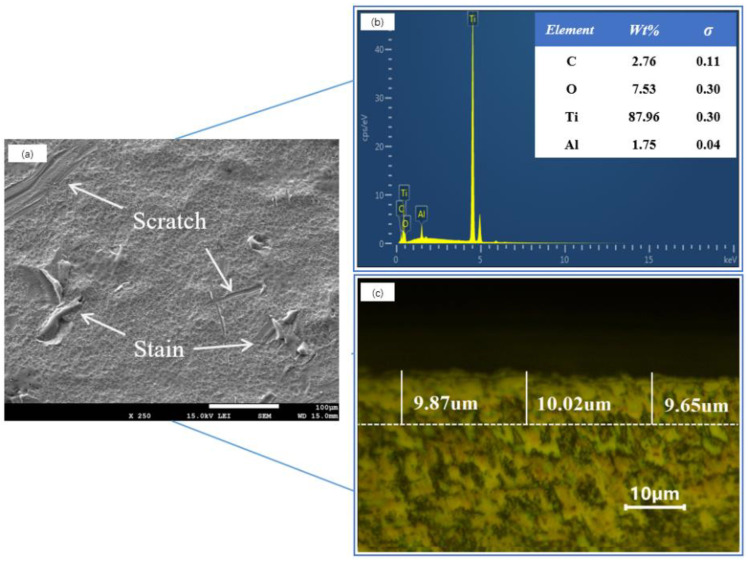
Micromorphology and composition of the TC4 titanium alloy oxide film: (**a**) SEM morphology at 250×; (**b**) EDS spectrum; and (**c**) thickness of the oxide film.

The chemical composition of the selected experimental material, TC4 titanium alloy, is listed in Table 1. Owing to its reactive chemical properties, the surface of the TC4 titanium alloy is easily oxidized in its natural state, forming a dense oxide film primarily composed of TiO_2_ [18]. The alloy was cut into 25 mm × 25 mm × 3 mm samples for cleaning experiments via a CNC wire-cutting machine (DK7735, Raygo Inc., Suzhou, China). The micromorphology of the TC4 titanium alloy and its surface elemental composition was measured via a scanning electron microscope (JSM-7610F, JEOL Inc., Tokyo, Japan), as shown in Figure 1a,b where σ denotes the standard deviation. The uncleaned samples covered with an oxide film contained contaminants and scratches, with numerous small oxide particles adhering to the surface of the oxide film. After the samples were embedded, the cross-sections were polished, and the oxide film area covering the substrate was observed via a metallurgical microscope (AE2000 MET, Motic Inc., Barcelona, Spain). The measured thickness of the oxide film was approximately 10 µm, as shown in Figure 1c.

### 2.2. Laser Cleaning Laboratory Equipment

**Figure 2:**
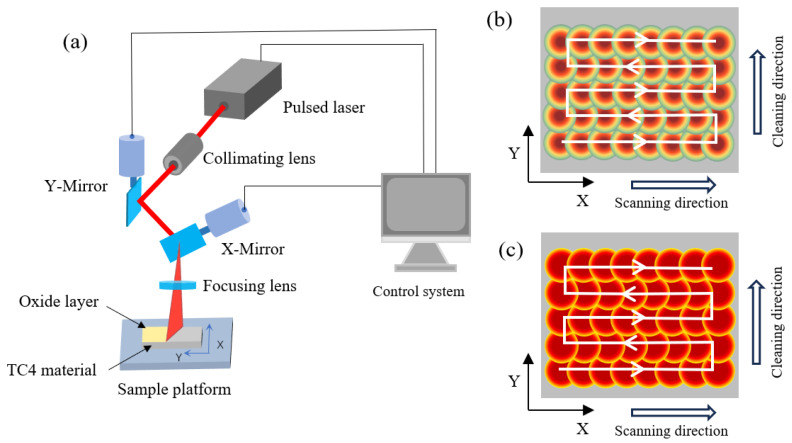
Laser cleaning system and scanning path; (**a**) laser cleaning system; (**b**) Gaussian spot scanning path; (**c**) Flat-top spot scanning path.

Figure 2a shows a schematic of the laser cleaning equipment, which consists mainly of a control system, a pulse laser, a scanning galvanometer system, and a worktable. The laser output is mounted on an industrial robot, traveling at a set speed in the y-direction. The scanning mirror oscillates at high speed in the x-direction, allowing the laser beam to pass through a focusing lens and irradiate the sample, enabling the laser cleaning of the oxide film on the TC4 titanium alloy surface. Figure 2b,c depict the scanning paths of laser beams with different spot patterns.

Table 2 key parameters of the Gaussian pulse laser (MFPT-300CL, MAX Inc., Shenzhen, China) and Flat-top pulse laser (MFPT-300CLS, MAX Inc., Shenzhen, China); the laser parameters are consistent between both devices.

The laser energy density is the main factor affecting the effectiveness of laser cleaning. According to the formula for the laser energy density *I*_0_ (J/cm^2^), the following is true [19]:

(1) $$I_{0}=\frac{2E_{P}}{\pi w_{0}^{2}}$$

Therefore, the design of single-factor experiments to analyze the effect of laser process parameters is shown in Table 3, where $E_{p}$ is the laser pulse energy (J), and $w_{0}$ is the beam radius (cm).

After laser cleaning, the surface micromorphology and elemental composition of TC4 were observed via a scanning electron microscope. A roughness-measuring instrument (SJ-140, MITUTOYO Inc., Kawasaki, Japan) was used to analyze the cleaned samples’ surface roughness. A comparative study of micromorphology, elemental composition, and roughness was conducted to evaluate the cleaning effectiveness of different spot pattern lasers.

### 2.3. Numerical Model

**Figure 3:**
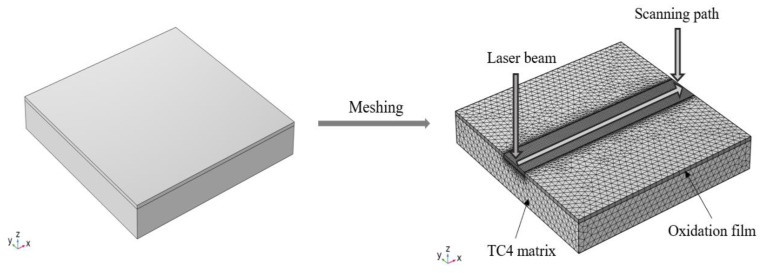
Geometry and mesh model for laser cleaning.

A layered model of TC4 titanium alloy and oxide film (primarily composed of TiO_2_) was established using COMSOL software, forming a composite structure. A significant temperature field gradient is generated since the laser directly acts on the oxide film region. To accurately simulate the variations in the temperature field, a combination of free tetrahedral and sweeping mesh methods was used, selecting ‘user-controlled mesh’ in the meshing module, as well as a hyperfine local mesh method, as shown in Figure 3, in which the number of cells is 181,904, the number of mesh vertices is 47,460, the total number of nodes in an element is 153,096, the number of degrees of freedom contained is 621,475, the number edge cells is 1100, the largest cell is 4 μm, the lowest cell mass is 0.1881, and the average cell mass is 0.6691. The thermal parameters of the oxide film are shown in Table 4, and the thermophysical parameters of TC4 were obtained from the material library built into the software.

Due to the complex physical processes involved in laser cleaning, including light, heat, and force interactions [20], the finite element model needs to be simplified as follows:

(1) The initial temperature of the model before laser cleaning is 293.15 K;
(2) The effects of temperature variation on the thermal properties of the materials are neglected;
(3) The materials are considered isotropic, with consistent physical properties in all directions;
(4) There is complete thermal conduction between the substrate and the cleaned material.

Based on the above assumptions, the heat transfer during laser cleaning follows Fourier’s law and the principle of energy conservation. The three-dimensional heat conduction control equation is given by the following [21]:

(2) $$\rho c\frac{\partial T}{\partial t}=\nabla (\kappa \nabla T)+\alpha (1-R)I\exp(-\alpha z)$$

During the simulation process, the following boundary conditions were applied [22,23,24].

The model exchanges heat with the environment, and the boundary conditions are as follows:

(3) $${\left.-\kappa \frac{\partial T}{\partial n}\right|}_{\Gamma }=h(T_{0}-T)$$

The heat convection on the boundaries of the model is as follows:

(4) $${\left.-\kappa \frac{\partial T}{\partial n}\right|}_{\Gamma }=\sigma \varepsilon (T_{0}^{4}-T^{4})$$

Under the irradiation of laser energy, the heat radiation at the boundaries of the model is as follows:

(5) $${\left.-\kappa \frac{\partial T}{\partial n}\right|}_{\Gamma }=Q-h(T_{0}-T)-\sigma \varepsilon (T_{0}^{4}-T^{4})$$

**Figure 4:**
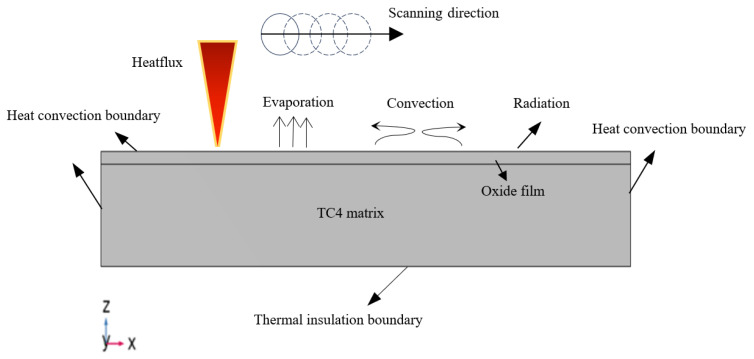
Schematic diagram of thermal boundary conditions.

The temperature field boundary conditions are shown in Figure 4. In the above equations, T(K) is the instantaneous temperature of the material; α is the absorption coefficient; ρ is the density of the material; c is the specific heat capacity of the material; κ is the thermal conductivity; R is the reflectivity; *I*(W·m^−2^) is the external heat source; h is the convective heat transfer coefficient; σ is the Stefan–Boltzmann constant; and ε is the surface emissivity.

**Figure 5:**
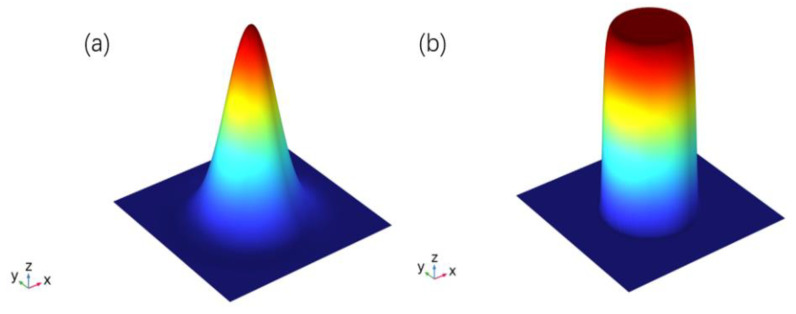
Energy distribution of laser spots: (**a**) Gaussian spot; (**b**) circular Flat-top spot.

The laser loading forms are categorized into Gaussian pulse and Flat-top pulse, as described by Equations (6) and (7) [25,26]. The energy distribution of the spot patterns is shown in Figure 5.

(6) $$Q=(1-R)I_{0}\exp(-2{\left(\frac{\left(x-x_{t}\right)}{r_{0}}\right)}^{2}-2{\left(\frac{\left(y-y_{t}\right)}{r_{0}}\right)}^{2})\cdot \varphi (t)$$

(7) $$Q=(1-R)I_{0}\exp(-2{\left(\frac{\left(x-x_{t}\right)}{r_{0}}\right)}^{n}-2{\left(\frac{\left(y-y_{t}\right)}{r_{0}}\right)}^{n})\cdot \varphi (t)$$

In the equations, *Q*(J/cm^2^) denotes the multi-pulse laser energy distribution function, n denotes the super-Gaussian exponent, which controls the steepness of the beam edges, $r_{0}$(cm) indicates the radius of the focused spot, and φ(t) represents the pulse function.

## 3. Results and Analysis

### 3.1. Simulation Results

**Figure 6:**
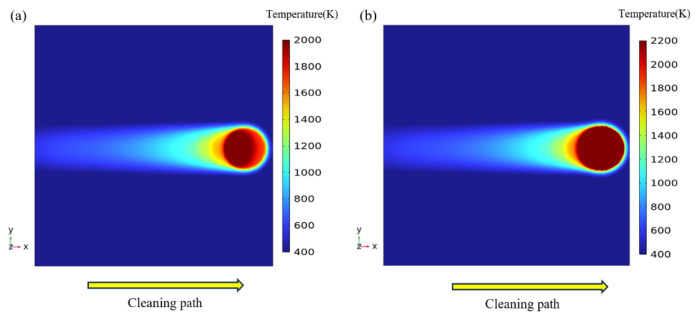
Temperature field distribution: (**a**) Gaussian pulse laser; (**b**) Flat-top pulse laser.

Figure 6 illustrates the temperature field distribution with different spot patterns during the laser cleaning. The temperature rises rapidly as the Gaussian and Flat-top pulse heat sources scan across the oxide film surface. Due to the rapid heating and cooling characteristics of pulsed laser heating, the scanning path retains a temperature field that has not yet cooled, creating a significant temperature gradient around the spot and forming the cleaning trajectory.

**Figure 7:**
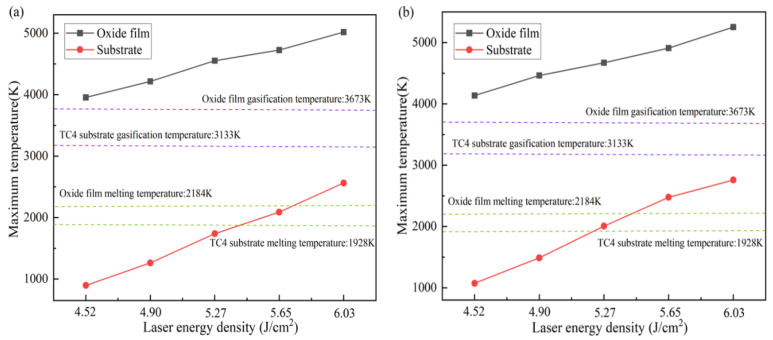
Temperature variation with time at different laser energy densities: (**a**) Gaussian pulse laser; (**b**) Flat-top pulse laser.

Figure 7a,b show the peak temperatures reached at the oxide film and substrate surface during the laser cleaning of a titanium alloy with different spot patterns. Under the same laser energy density output, the Gaussian spot concentrates energy at the center while gradually diminishing at the edges. In contrast, the Flat-top spot achieves a higher and more uniform energy distribution. As a result, the peak temperature reached during Flat-top spot cleaning is higher than that of the Gaussian spot. When the laser energy density is 4.52 J/cm^2^, the temperature of the oxide film surface exceeds its vaporization temperature (3673 K), initiating the removal of the oxide film. At an energy density of 5.27 J/cm^2^, the substrate surface temperature approaches its melting point (1928 K), indicating that the laser begins to act on the exposed substrate surface. As the laser energy density increases, the substrate absorbs more, potentially causing varying degrees of damage to the material.

**Figure 8:**
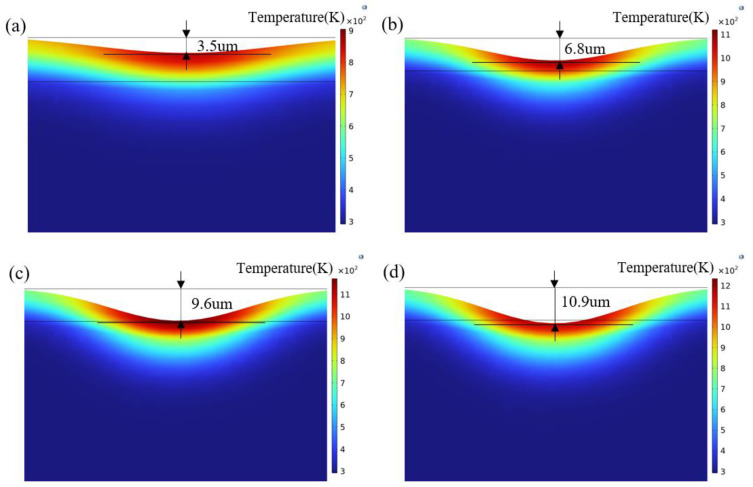
Ablation depth of Gaussian pulse laser: (**a**) 4.52 J/cm^2^; (**b**) 4.90 J/cm^2^; (**c**) 5.27 J/cm^2^; (**d**) 5.65 J/cm^2^.

When the laser scanning speed is set at 6000 mm/s, the ablation depths of the Gaussian pulse laser at different energy densities are shown in Figure 8a–d. The pit shape is related to the energy distribution of the Gaussian pulse laser, with the deepest ablation occurring at the center of the spot, gradually becoming more shallow as it moves away from the center. As the laser energy density increases, the impact of laser energy on the temperature field of the oxide film also grows, leading to continuous energy accumulation in the ablation depth direction. At a laser energy density of 5.27 J/cm^2^, the simulation results indicate effective removal of the oxide film. When the energy density increases to 5.65 J/cm^2^, the ablation depth reaches 10.9 µm, intensifying the laser ablation effect and beginning to affect the substrate.

**Figure 9:**
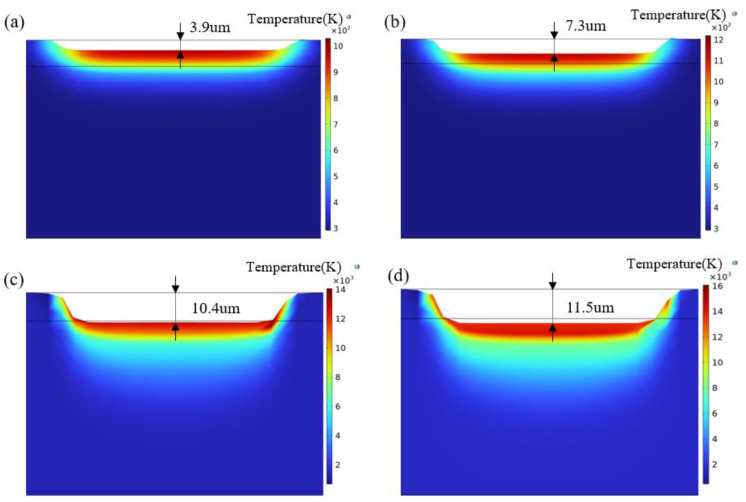
Ablation depth of Flat-top pulse laser: (**a**) 4.52 J/cm^2^; (**b**) 4.90 J/cm^2^; (**c**) 5.27 J/cm^2^; (**d**) 5.65 J/cm^2^.

Figure 9a–d show the ablation depths of the Flat-top pulse laser at different energy densities. Due to the characteristics of the Flat-top spot, it can be observed that the bottoms of the ablated pits are relatively flat. The variation in ablation depth is fundamentally similar to that of the Gaussian laser; however, since the Flat-top pulse laser outputs energy more uniformly, the heat-affected zone is more extensive, resulting in a more significant amount of thermal output to the material. Consequently, the temperature in the temperature field and the ablation depths are greater than those of the Gaussian pulse laser. At a laser energy density of 5.27 J/cm^2^, the oxide film is nearly completely removed, but the ablation depth reaches 10.4 µm, indicating that the laser has begun to affect the substrate surface. When the laser energy density is increased to 5.65 J/cm^2^, the ablation depth reaches 11.5 µm. At this point, the accumulation of laser energy is significant, causing damage to the substrate and resulting in poorer cleaning effectiveness.

### 3.2. Microstructural Analysis

**Figure 10:**
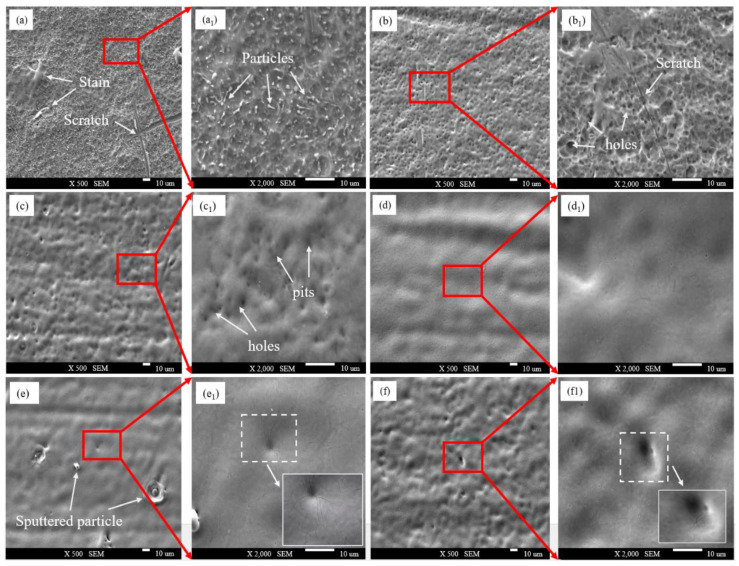
Microstructure of Gaussian pulse laser at different laser energy densities. (**a**,**a1**) Before cleaning; (**b**,**b1**) 4.52 J/cm^2^; (**c**,**c1**) 4.90 J/cm^2^; (**d**,**d1**) 5.27 J/cm^2^; (**e**,**e1**) 5.65 J/cm^2^; (**f**,**f1**) 6.03 J/cm^2^.

**Figure 11:**
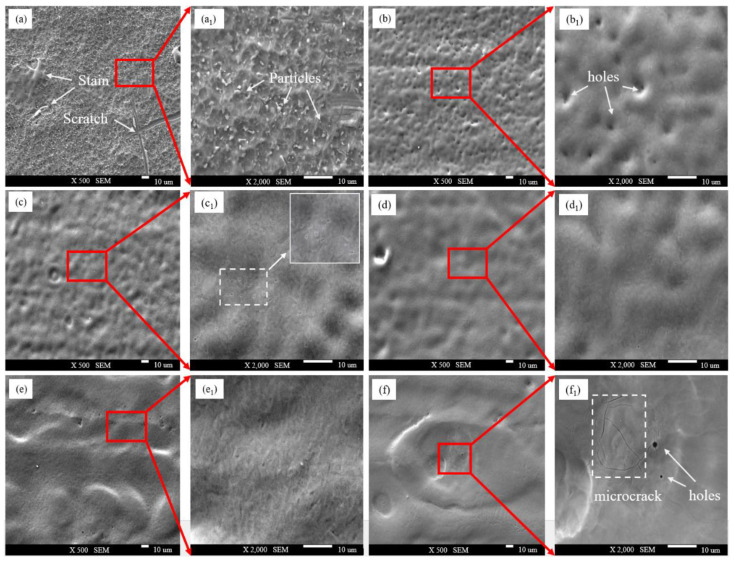
Microstructure of Flat-top pulse laser at different laser energy densities: (**a**,**a1**) Before cleaning; (**b**,**b1**) 4.52 J/cm^2^; (**c**,**c1**) 4.90 J/cm^2^; (**d**,**d1**) 5.27 J/cm^2^; (**e**,**e1**) 5.65 J/cm^2^ (**f**,**f1**) 6.03 J/cm^2^.

To compare the removal effects of different spot pattern lasers on the oxide film, following laser cleaning, the microstructure of the TC4 titanium alloy oxide film was examined using SEM. Figure 10a and Figure 11a show the microstructure before cleaning, revealing a significant amount of fine oxide particles adhering to the surface of the oxide film. Figure 10b–f and Figure 11b–f display the microstructures after cleaning with Gaussian and Flat-top pulse lasers, respectively, with (a1) to (f1) representing corresponding high-magnification images of specific areas.

During the cleaning process with a Gaussian pulse laser, at 4.52 J/cm^2^, only surface oxide particles and some of the oxide film was removed, leaving an uneven, porous original morphology. The holes formed when the oxide film melted, and the air was released from the pits, solidifying quickly afterward. At 4.90 J/cm^2^, the oxide removal rate improved, and the number of holes significantly decreased; however, some oxide film remained on the substrate due to insufficient laser energy. At 5.27 J/cm^2^, the thermal input to the substrate increased, resulting in the near-complete removal of the oxide film, with a relatively smooth and clean substrate surface, free of residual impurities. When the energy density increased from 5.65 J/cm^2^ to 6.03 J/cm^2^, the thermal accumulation effect intensified, transferring excess heat to the substrate. Once the temperature reached the melting point, the substrate underwent a solid–liquid phase change, leading to shallow traces of laser ablation, melting, spattering, and solidification on the surface, along with microcracks formed due to surface tension after the rapid cooling of the molten material [27].

During the cleaning process with a Flat-top pulse laser, the surface oxide particles were removed at 4.52 J/cm^2^, causing the surface oxide film to melt and form holes as it quickly solidified. At 4.90 J/cm^2^, the overall morphology improved, with most of the oxide film removed and micro-holes forming on the surface. At 5.27 J/cm^2^, the thermal accumulation effect intensified, leading to the near-complete removal of the oxide film, with the substrate surface slightly remelting, further enhancing its smoothness. When the laser energy density increased from 5.65 J/cm^2^ to 6.03 J/cm^2^, the laser energy was sufficient to remove the surface oxide film. However, the remaining laser energy continued to act on the titanium alloy surface, causing the shallow melting of the substrate. Under the strong impact of the Flat-top pulse laser, the molten surface material was dispersed. The rapid cooling of the material and the resulting surface tension created an unordered remelted structure, leading to microcracks and varying degrees of damage to the substrate surface, which resulted in decreased smoothness. This was generally consistent with the simulation results, validating the reasonableness of the numerical modeling.

### 3.3. Removal Mechanism Analysis

**Figure 12:**
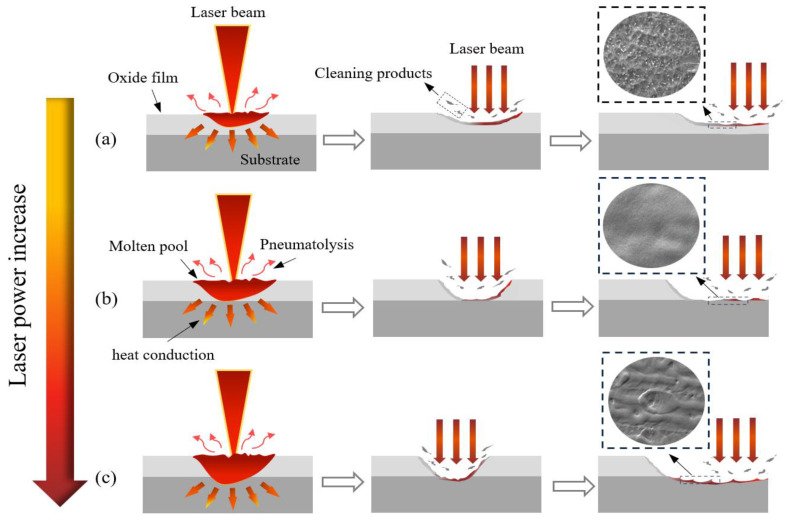
Schematic of laser cleaning of TC4 titanium alloy oxide film. (**a**) low fluence; (**b**) appropriate fluence; (**c**) high fluence.

The cleaning effects of different spot patterns are similar. Still, there are differences in the removal mechanisms of the TC4 titanium alloy oxide film at various laser energy densities, resulting in different surface microstructures. This section further explores the removal mechanisms observed during the cleaning process in conjunction with the microstructure of the sample surface. The oxide film absorbs laser energy at lower laser density and rapidly heats up. Upon reaching the melting point (2184 K), it begins to melt; however, due to the low energy absorbed, only slight melting occurs, leaving shallow pits on the surface, as shown in Figure 12a. When an appropriate laser energy density is applied, the absorbed laser energy increases, raising the temperature until melting and vaporization occur, resulting in the effective removal of the oxide film and a relatively smooth substrate surface, as depicted in Figure 12b. At higher laser energy density, while the oxide film is removed, excessive thermal accumulation damages the substrate. The molten substrate surface is influenced by both the laser impact and its surface tension. With rapid temperature decrease, it quickly solidifies, forming an unordered remelted structure, as shown in Figure 12c. Thus, the laser cleaning of the titanium alloy oxide film involves the surface absorbing significant laser energy. When the surface reaches its boiling point, cleaning occurs through evaporation and spattering, indicating that ablation and vaporization primarily drive the removal mechanism.

### 3.4. Surface Element Analysis

**Figure 13:**
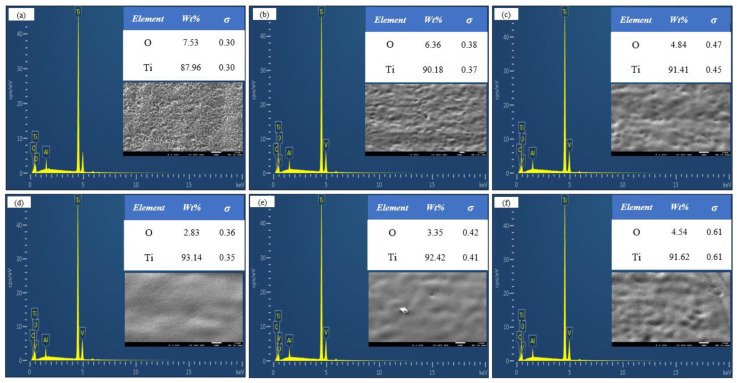
Energy spectrum analysis after Gaussian pulse laser cleaning. (**a**) Before laser cleaning; (**b**) 4.52 J/cm^2^; (**c**) 4.90 J/cm^2^; (**d**) 5.27 J/cm^2^; (**e**) 5.65 J/cm^2^; (**f**) 6.03 J/cm^2^.

The elemental composition was analyzed using an energy-dispersive spectrometer following laser cleaning to examine further the impact of various laser spot modes on the cleaning efficacy of the TC4 titanium surface oxide coating. Since the main components of the TC4 titanium alloy surface oxide film are oxides, the changes in oxygen and titanium content can more effectively reflect the degree of oxide film removal. The measured contents of oxygen and titanium in the uncleaned sample are shown in Figure 13a, which are 7.53% and 87.96%, respectively.

Figure 13b–f show the elemental content of the surface after cleaning with Gaussian pulse lasers at different energy densities. The mass percentage of titanium rises from 90.18% to 93.14%, whereas the mass percentage of oxygen falls from 6.36% to 2.83% as the laser energy density rises from 4.52 J/cm^2^ to 5.27 J/cm^2^. This indicates that appropriately increasing the laser energy density can effectively remove the oxide film, thereby increasing the exposed area of the titanium alloy substrate. At an average laser power of 5.65 J/cm^2^, the mass percentage of oxygen increases to 3.54%, and the mass percentage of titanium decreases to 91.62%. This aligns with the results from Section 3.2, indicating that while the oxide film is being removed, the substrate surface absorbs excessive heat, causing the material to melt and form new oxides upon contact with air, increasing oxygen content. With the laser energy density reaching 6.03 J/cm^2^, the thermal oxidation reaction intensifies, further increasing the mass percentage of oxygen to 4.54%.

**Figure 14:**
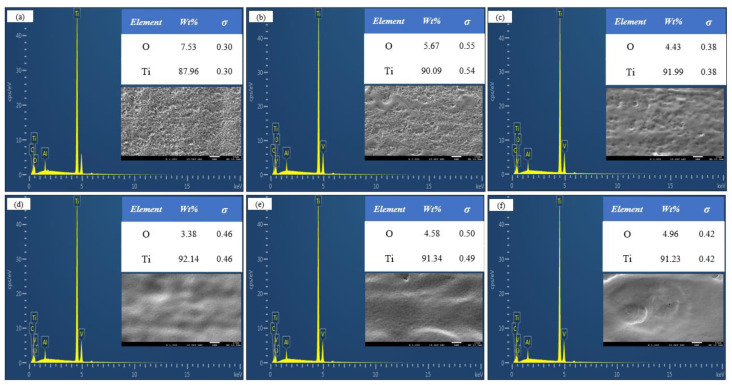
EDS analysis after cleaning with Flat-top pulse laser. (**a**) Before laser cleaning; (**b**) 4.52 J/cm^2^; (**c**) 4.90 J/cm^2^; (**d**) 5.27 J/cm^2^; (**e**) 5.65 J/cm^2^; (**f**) 6.03 J/cm^2^.

Figure 14a–f illustrate the elemental composition after cleaning with Flat-top pulsed lasers at different energy densities. When the laser energy density increases from 4.52 J/cm^2^ to 5.27 J/cm^2^, the oxide film is effectively removed, decreasing the oxygen mass percentage to 3.38% and the titanium mass percentage to 92.14%. At 5.65 J/cm^2^, the oxygen mass percentage rises again to 4.58%, while the titanium mass percentage decreases to 91.34%. This analysis is consistent with previous results: while the oxide film is removed, the residual energy continues acting on the substrate surface. Due to the high temperature of the laser and reactions with oxygen atoms in the air, a new thermal oxide layer is formed, and the remaining laser energy is insufficient to eliminate this newly formed oxide. At a laser energy density of 6.03 J/cm^2^, the reaction between the molten material and atmospheric oxygen intensifies, causing the oxygen mass percentage to increase to 4.96%.

**Figure 15:**
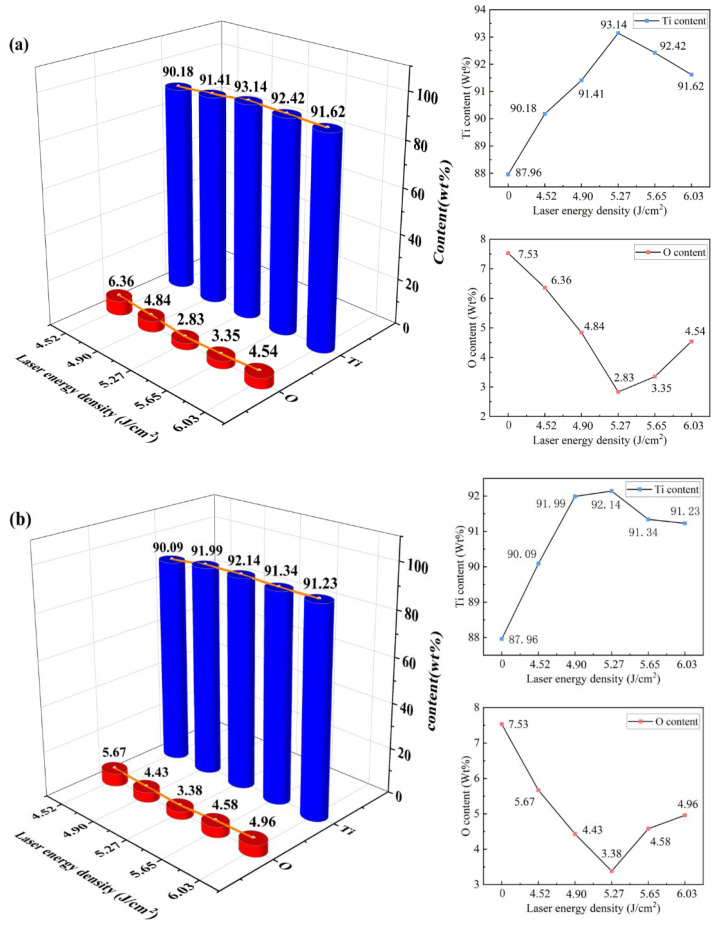
Trends in elemental content variation. (**a**) Gaussian pulse laser; (**b**) Flat-top pulse laser.

Combining the above analyses, as the laser energy density increases, the overall trend for oxygen elements shows a decrease followed by a rise. In contrast, the titanium elements exhibit a corresponding increase followed by a decline, as shown in Figure 15a,b. When the laser energy density is between 4.52 and 4.90 J/cm^2^, the oxygen content after cleaning with a Flat-top pulse laser is lower than that after cleaning with a Gaussian pulse laser. This is attributed to the more uniform energy output of the Flat-top pulse laser, which enhances the ablation effect on the oxide layer. However, when the laser energy density increases to between 5.27 and 6.03 J/cm^2^, there is sufficient laser energy to remove the oxide layer, and the remaining laser energy continues to act on the substrate surface, leading to thermal oxidation reactions. Due to the larger heat-affected zone produced by the Flat-top pulse laser on the substrate surface, the thermal oxidation reactions become more intense, resulting in the oxygen content exceeding that of the Gaussian pulse laser after cleaning with the Flat-top pulse laser.

### 3.5. Surface Roughness Analysis

**Figure 16:**
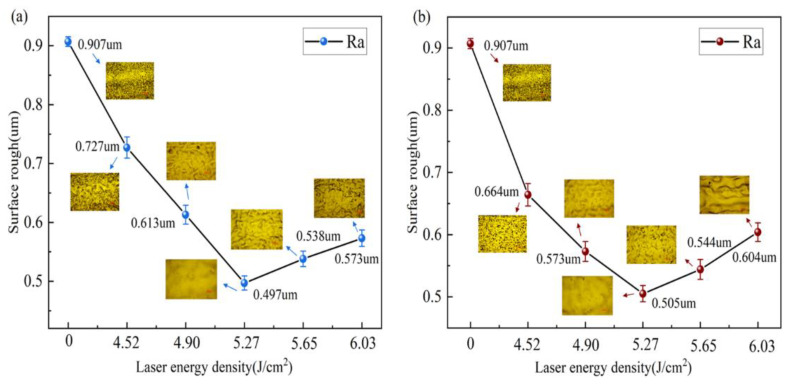
Surface Ra values under different laser energy densities. (**a**) Gaussian pulse laser; (**b**) Flat-top pulse laser.

The machinability and corrosion resistance of materials are significantly influenced by surface roughness [28]. Therefore, studying the surface roughness of materials is of significant importance. Before cleaning, the material surface exhibited numerous pits and scratches, with a Ra value of approximately 0.907 µm. After cleaning the TC4 titanium alloy with different laser spot patterns, as shown in Figure 16a,b, it can be observed that as the laser energy density increases, the Ra value initially decreases and then increases. At relatively low laser energy densities, the oxidation film undergoes remelting, resulting in a smoother surface compared to the uncleaned state, with reduced roughness. The cleaning effect is at its best when the Gaussian pulse laser’s energy density hits 5.27 J/cm^2^, achieving a minimum Ra value of 0.497 µm. As the laser energy density continues to increase to 5.65 J/cm^2^–6.03 J/cm^2^, the increased pulse energy causes thermal damage to the substrate surface, leading to greater fluctuations and the formation of melt and microcracks, with Ra values rising to 0.538 µm and 0.573 µm, respectively. The surface does not exhibit significant fluctuations for the Flat-top pulse laser at an energy density of 5.27 J/cm^2^, with a minimum Ra value of 0.505 µm, indicating effective cleaning. However, when the laser energy density increases to 5.65 J/cm^2^–6.03 J/cm^2^, excessive cleaning occurs, resulting in surface melting and disordered movement due to plasma shock waves, leading to different degrees of disordered remelted structures after cooling, with Ra values increasing to 0.544 µm and 0.604 µm, respectively.

In summary, when the laser energy density is between 4.52 J/cm^2^ and 4.90 J/cm^2^, the laser acts on the substrate surface’s oxidation film, improving surface flatness. Due to the more uniform energy output of the Flat-top pulse laser, its ablation effect on the oxidation film is more pronounced, resulting in a lower Ra value compared to Gaussian pulse laser cleaning. At 5.27 J/cm^2^, the Ra values reach their minimum, indicating that the oxidation film has mainly been removed, while the substrate experiences slight remelting, resulting in a polishing effect. However, when the energy density increases to 5.65 J/cm^2^–6.03 J/cm^2^, the Flat-top pulse laser generates greater laser impact on the material surface and the driving force of surface tension, leading to increased fluctuations, resulting in higher Ra values than those observed with Gaussian pulse laser cleaning.

## 4. Conclusions

(1) A finite element model for laser cleaning with different spot patterns was established. As the laser energy density increased, the maximum temperatures of the oxide layer and substrate surfaces rose, leading to a significant increase in ablation depth. The temperature and removal depth achieved with Flat-top pulse lasers were greater than those with Gaussian pulse lasers, which were verified through experiments.
(2) Under different spot pattern laser cleaning conditions, significant oxide layer removal occurred when the laser energy density was between 4.52 J/cm^2^ and 5.27 J/cm^2^. When the energy density increased from 5.65 J/cm^2^ to 6.03 J/cm^2^, melting damage to the substrate was observed. The damage level from Gaussian pulse laser cleaning was lower than that from Flat-top pulse laser cleaning, with the removal mechanism primarily based on ablation and vaporization.
(3) Within the laser energy density range of 4.52 J/cm^2^ to 6.03 J/cm^2^, the content of oxygen (O) first decreased and then increased, while the changes in titanium (Ti) content exhibited the opposite trend. At a laser energy density of 5.27 J/cm^2^, the O content dropped to its lowest at 2.83%, while Ti content was at 3.38%, indicating better cleaning performance with Gaussian pulse lasers.
(4) Surface roughness was primarily influenced by morphological changes, showing a trend of first decreasing and then increasing with rising laser energy density. At a laser energy density of 5.27 J/cm^2^, the Ra value reached its minimum, measuring 0.497 μm for Gaussian pulse lasers and 0.505 μm for Flat-top pulse lasers, with the latter being slightly higher.
(5) During the oxide layer cleaning phase, the surface temperatures achieved with Flat-top pulse lasers were higher, leading to better cleaning effects. However, when the oxide layer was mainly removed, Gaussian pulse lasers offered easier control over cleaning effects and depth, resulting in less damage.

## Figures and Tables

**Table 1:** Chemical composition of TC4 titanium alloy.

Element	Ti	Al	V	Fe	C	N	H	O
Content/wt%	Bal.	5.66	3.75	0.30	0.08	0.05	0.015	0.20

**Table 2:** Main technical parameters of Gaussian pulse and Flat-top pulse lasers.

Parameter	Value
Wavelength/nm	1064
Average Power/W	≤300
Pulse Width/ns	≤500
Frequency/kHz	≤500
Scan Speed/mm⋅s^−1^	≤20,000
Spot Diameter/um	130

**Table 3:** Laser process parameters.

Parameter	Value
Energy Density/J⋅cm^−2^	4.52, 4.90, 5.27, 5.65, 6.03
Pulse Width/ns	110
Frequency/kHz	300
Scan Speed/mm⋅s^−1^	6000

**Table 4:** Thermal parameters of oxide film.

Parameter	Value
Density *ρ*/(kg·m^−3^)	4280 − 0.06 × T − 7.69 × 10^−5^ × T^2^ + 4.50 × 10^−8^ × T^3^ −8.76 × 10^−2^ × T^4^, 273 K ≤ T ≤ 2023 K; 4060, 2023 K < T;
Specific heat capacity *c*/(J·kg^−1^·K^−1^)	−9,743,633 × T^−2^ + 553.7 + 0.188 × T, 293 K < T < 1264 K;
	620.76 + 0.157 × T, 1264 K ≤ T ≤ 1800 K; 903.64, 1800 K < T;
Thermal conductivity *κ*/(W·m^−1^·K^−1^)	31.02 − 0.13 × T + 2.719 × 10^−4^ × T^2^ − 2.71 × 10^−7^ × T^3^ + 1.32 × 10^−10^ × T^4^ − 2.55 × 10^−14^ × T^5^, 293 K ≤ T ≤ 1273 K; 3.30, 1273 K < T;
+Melting temperature T_m_/K	2184
Boiling temperature T_b_/K	3673
Laser absorptivity α	0.6
Reflectivity R	0.55
Surface emissivity *ε*	0.58
Stefan–Boltzmann constant *ρ*/(W·m^−2^·K^−4^)	5.67 × 10^−8^
Thermal convection coefficient h/(W·m^−2^·K^−1^)	10

## Data Availability

The original contributions presented in the study are included in the article, further inquiries can be directed to the corresponding author.
